# Voriconazole-induced periostitis in the hand

**DOI:** 10.1016/j.radcr.2024.11.079

**Published:** 2024-12-19

**Authors:** Gurbinder Singh, Daria Motamedi, Kevin Sweetwood

**Affiliations:** a School of Medicine, University of California-San Francisco, 533 Parnassus Ave, San Francisco, CA, USA; b Department of Radiology, University of California-San Francisco, 513 Parnassus Ave, Room S257, Box 0628, San Francisco, CA 94143, USA

**Keywords:** Voriconazole, Periostitis, Drug reaction, Radiology, Bone pain, Hand

## Abstract

Voriconazole, a triazole antifungal, has proven effective against invasive fungal infections, and is often selected due to its enhanced antifungal spectrum coverage. Despite its general tolerability, voriconazole usage is associated with drug-induced periostitis, which presents with diffuse bone pain. This case report details a 65-year-old male on chronic immunosuppressive and antimicrobial therapy following heart transplant who developed hand pain. Identification of periostitis on hand radiographs and an acute elevation of alkaline phosphatase on subsequent blood analysis prompted discontinuation of voriconazole, resulting in a symptomatic and laboratory improvement. This case underscores the importance of leveraging a thorough understanding of imaging features, clinical manifestations, and laboratory data to aid in the diagnosis of voriconazole-induced periostitis.

## Introduction

Voriconazole, a triazole antifungal agent hepatically metabolized via cytochrome P450, has demonstrated efficacy against invasive fungal infections, and is often selected for its extended coverage, including invasive aspergillosis, and more effective inhibition of 14-α-sterol demethylase than other azoles [[1], [2], [3]]. While voriconazole offers broad antifungal coverage, it is important to recognize the potential for serious adverse effects, particularly periostitis, which can develop with prolonged use and higher dosages. The pathology of voriconazole-induced periostitis is thought to mirror that of fluorosis given the high quantity of fluorine within the medication, leading to increased incorporation of fluorapatite deposition in the osseous extracellular matrix [4].The risk of developing this complication increases with cumulative dose and duration, likely related to the increased exposure to fluoride [4]. Diagnosis of voriconazole-induced periostitis typically involves recognizing diffuse or localized bone pain, elevated bone-specific alkaline phosphatase levels, and characteristic imaging findings such as periosteal reaction and cortical thickening [4,5]. Prompt diagnosis is essential as discontinuation of the medication provides rapid symptomatic relief [6].This report details an instance of voriconazole-associated periostitis in a postheart transplant patient, with observed symptomatic, laboratory, and radiologic improvement following the discontinuation of the medication.

## Case report

A 65-year-old male with a prior medical history of severe coronary artery disease and ischemic cardiomyopathy status post heart transplant and immunosuppressive therapy presented with a violaceous nodular foot lesion, subsequently identified as Medicopsis remoeri on biopsy.

The biopsy results prompted initiation of voriconazole antifungal therapy (400 mg BID for 1 day, followed by 200 mg BID). After 1 week of starting treatment, his voriconazole trough level was 6.4 μg/mL (normal limits 1.0–5.5 μg/mL), prompting a reduction in his voriconazole dosage to 150 mg BID. His next trough, approximately 2.5 months after starting therapy, was 1.7 μg/mL. Of note, the patient was known to be a CYP3A5 poor metabolizer.

**Fig. 1:**
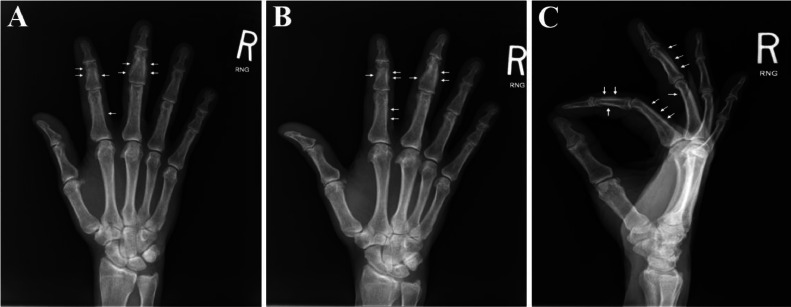
Anterio-posterior (A), Oblique (B), Lateral (C).

Approximately 5 months after initiating voriconazole, the patient reported newly developed hand pain. A radiograph was ordered which demonstrated multifocal well-mineralized periosteal reaction about the second and third middle phalanges and, to a lesser extent, the proximal phalanges with “fluffy” morphology (Fig. 1), which in the provided clinical context raised concern for voriconazole-induced periostitis. Infection, hypertrophic osteoarthropathy, or trauma were considered less likely due to multifocality, distribution of involvement, morphology of the periosteal reaction, and absence of bone loss or deformity. Subsequent blood analysis revealed acutely elevated alkaline phosphatase (202 U/L, reference range 38-108 U/L).

Multifocal periosteal reaction involving the second and third middle phalanges and to a lesser extent of the proximal phalanges. The periosteal reaction demonstrates well-mineralized and “fluffy” appearance. No associated erosive changes, bone loss, or posttraumatic deformity. No additional sites of periosteal reaction or lytic/sclerotic lesions.

Voriconazole was immediately discontinued with subsequent symptomatic improvement and normalization of alkaline phosphate levels after 2 months.

## Discussion

Voriconazole, a fluorine-containing antifungal agent, is known to cause periostitis through mechanisms similar to fluorosis [7]. The bone extracellular matrix, primarily composed of hydroxyapatite, can incorporate fluoride to form fluorapatite when fluoride levels are high. Fluorapatite increases bone matrix density and resistance to resorption, leading to osteosclerosis [7]. This increased bone density results in brittleness, decreased mechanical competence, and a higher susceptibility to fractures. Fluorapatite also stimulates osteoblastic activity, further inducing periostitis and exostoses [8].

Voriconazole is metabolized via the CYP2C19, 2C9, and 3A4 enzymes, and polymorphisms in these enzymes can affect drug metabolism and plasma concentrations, impacting drug efficacy and safety [9,10]. Most cases of voriconazole-induced periostitis occur in immunocompromised individuals, such as those on chronic immunosuppression post-transplantation, due to the need for higher cumulative doses for fungal therapy [11,12].

Voriconazole-induced periostitis typically presents with diffuse or localized osteoarticular pain involving the appendicular skeleton, ribs, and scapulae [13]. Elevation of bone-specific alkaline phosphatase is the most common laboratory abnormality of periostitis [14], and is typically observed in patients receiving higher doses of voriconazole [14,15]. Radiographs are the first-line imaging technique and often show multiple areas of inflammation along the outer layer of bones (periosteum), which can lead to new bone formation. These new bone formations can appear in different patterns, such as uneven or patchy growths, soft feather-like edges, or small, rounded bumps, distributed unevenly across the bone surfaces [16,17]. Technetium 99m-methyl diphosphonate scintigraphy can also be utilized, which demonstrates increased tracer uptake corresponding to the regions of periosteal reaction [18]. Advanced imaging modalities such as MRI are not typically necessary for diagnosis, however can reveal new periosteal bone formation with elevated linear hypointense periosteum surrounded by various degrees of T2 hyperintense peri-cortical soft tissue and/or bone marrow edema-like signal [19,20].

Moon W et al. reported a significant difference in daily voriconazole doses between patients with periostitis (780 ± 43 mg/day) and those without periostitis (400 mg/day) [15]. In this case, periostitis occurred at a lower dose of 150 mg/day, which raises questions about the patient's underlying metabolic factors. The patient's status as a CYP3A5 poor metabolizer likely played a critical role, as CYP3A5 is a key hepatic enzyme in the metabolism of voriconazole [21].

Individuals who are poor metabolizers have reduced or absent CYP3A5 enzyme activity, leading to higher systemic levels of voriconazole when given standard doses [22]. This can increase the risk of adverse effects, such as periostitis, even at lower dosages that would typically be considered safe for those with normal metabolism [23]. In this context, the patient's genetic variation in CYP3A5 may have heightened the drug's effects, making them more susceptible to bone toxicity. As a result, prolonged drug exposure due to reduced enzyme activity could have led to toxic accumulation, increasing the risk of developing periostitis, even while the patient maintained stable trough levels.

This case report of voriconazole-induced periostitis is noteworthy as it challenges conventional understanding that cumulative dose and trough levels reliably predict periostitis risk. Moreover, our report highlights a unique presentation of voriconazole-induced periostitis isolated to the hand, broadening the spectrum of documented cases. These findings emphasize that clinicians should consider periostitis risk even at lower voriconazole doses and highlight the need for individualized management. Early recognition of periostitis, regardless of dose, is crucial. Routine monitoring strategies should include clinical assessments to evaluate diffuse bone pain—often severe and disabling—particularly in the hands, arms, legs, and shoulders. Laboratory tests revealing elevated alkaline phosphatase and serum fluoride levels are also characteristic of periostitis and should be continually monitored. X-rays are essential for diagnosis, while advanced imaging like MRI and PET scans can be utilized if the diagnosis remains unclear. Further research and documentation are essential to explore how different doses may trigger periostitis and to enhance awareness of this rare complication.

## Patient consent

Written informed consent was obtained from the patient and/or their family for the publication of this case report. The patient and/or their family has been made aware that their medical information will be published, and they consented to its inclusion in this article.
